# Melanocytic matricoma: a rare tumor that can mimic melanoma^⋆^

**DOI:** 10.1016/j.abd.2022.08.001

**Published:** 2022-09-07

**Authors:** Iñigo Aranguren-López, Sara Ibarbia-Oruezabal, Nerea Segués-Merino

**Affiliations:** a Department of Dermatology, Hospital Universitario Donostia, San Sebastián, Spain; b Department of Pathology, Hospital Universitario Donostia, San Sebastián, Spain

Dear Editor,

Melanocytic matricoma is a rare benign cutaneous adnexal tumor that recapitulates the early anagen hair follicle.^1^ It was first described in 1999, and to date, there are approximately 20 published cases in the literature.^2^

A 60-year-old man with HIV under antiretroviral therapy presented with a 2-month history of a rapidly growing papule affecting his left preauricular area. Physical examination showed a well-defined 6 mm papule with an uneven coloration ranging from pink to grey (Fig. 1). Complete surgical excision of the tumor was performed. Histopathology revealed a well-circumscribed, multinodular, asymmetrically pigmented dermal tumor, with no connection to the overlying epidermis. It was composed of basaloid cells with scant cytoplasm and distinct nucleoli, showing moderate pleomorphism and 10 mitoses per 10 high-power fields. Occasional aggregates of shadow cells were also observed. Heavily pigmented dendritic melanocytes without atypia were scattered among basaloid cells, forming small clusters. There was no necrosis or calcification in the tumor (Fig. 2). Basaloid cells were positive for cytokeratin AE1/AE3, cytokeratin 5/6, and beta-catenin, and the melanocytic component was highlighted by HMB-45 (Fig. 3). These findings were consistent with the diagnosis of melanocytic matricoma. There is no evidence of local recurrence or metastasis 26 months after excision.

**Figure 1:**
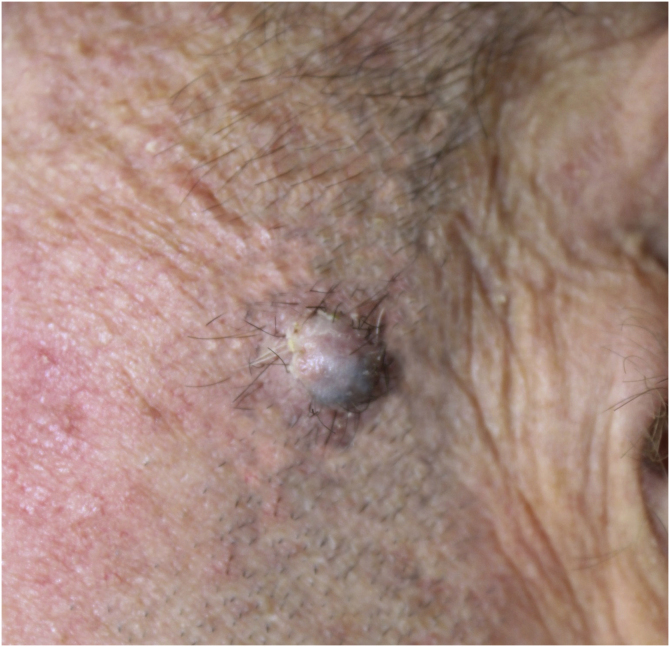
A well-defined greyish-pink papule on the left preauricular area.

**Figure 2:**
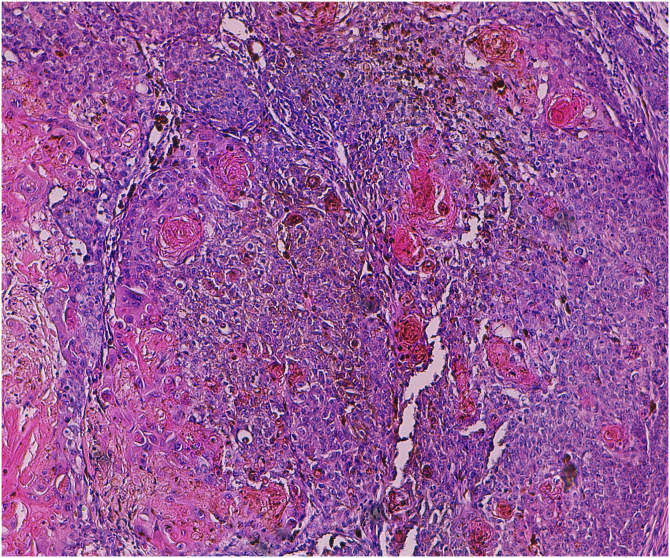
Histopathology of the tumor. The tumor is composed predominantly of basaloid cells with small clusters of eosinophilic shadow cells, as well as heavily pigmented melanocytes interspersed among the epithelial component (Hematoxylin & eosin, ×100).

**Figure 3:**
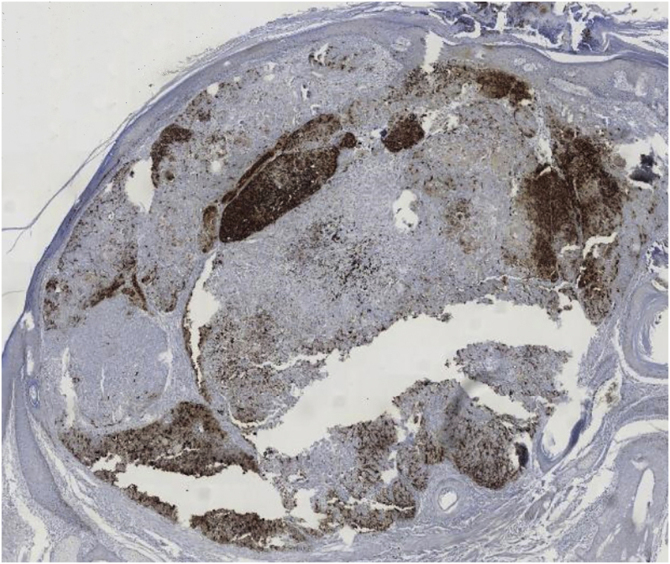
Immunohistochemistry. HMB45 staining highlights dendritic melanocytes forming clusters (original magnification ×40).

Melanocytic matricoma typically presents as a sharply demarcated, small (generally smaller than 1 cm) unevenly pigmented papule or nodule, arising on sun-damaged skin of elderly individuals, with a male predominance.^3^, ^4^ Histologically, it is a well-circumscribed nodular or multinodular dermal biphasic neoplasm, composed of epithelial cells with matrical differentiation and melanocytes. The epithelial component consists of basaloid matrical and supramatrical cells, which are mild to moderately pleomorphic and mitotically active.^3^ Small aggregates of eosinophilic shadow cells can also be present.^4^ Dendritic melanocytes without atypia are scattered among epithelial cells and show strong pigmentation.^3^ The epithelial component shows positivity for cytokeratin and beta-catenin, whereas dendritic melanocytes are highlighted by HMB-45, S-100 and Melan-A.^3^ Cases with atypical histologic features have been reported, such as melanocytic atypia, epidermal connection, calcification, granulomatous inflammation, epidermal consumption, or cystic degeneration.^1^, ^2^, ^3^, ^4^ None of these features were present in the present case.

Clinical differential diagnosis of this entity includes malignant melanoma, pigmented Basal Cell Carcinoma (BCC), and hemangioma.^3^ Histopathological differential diagnosis includes tumors with matrical differentiation, such as pilomatricoma with melanocytic hyperplasia, malignant pilomatricoma, matricoma, BCC with matrical differentiation^3^ and, most challengingly, malignant melanocytic matricoma, an extremely rare tumor with only 8 reported cases.^5^ Proposed criteria for differentiating benign and malignant melanocytic matricoma remain controversial^2^, ^5^; recurrence, metastasis, necrosis, ulceration, an infiltrative growth pattern with pushing borders, and marked cytological atypia would suggest malignancy,^1^, ^5^ but according to some authors, a high mitotic rate would not constitute a reliable criterion of aggressive behavior.^5^

Despite being considered a benign tumor, the clinical behavior of melanocytic matricoma remains unknown because of the few reported cases and the lack of long-term follow-up.^1^, ^4^, ^5^ Therefore, complete surgical excision and periodic re-examinations are recommended.^1^, ^5^ Dermatologists and dermatopathologists should be aware of this rare entity when facing a pigmented lesion with a dual epithelial/melanocytic component to avoid misdiagnosis.^1^, ^5^

## Financial support

None declared.

## Authors’ contributions

Iñigo Aranguren-López: Approval of the final version of the manuscript; design and planning of the study; drafting and editing of the manuscript; collection, analysis, and interpretation of data; effective participation in research orientation; intellectual participation in the propaedeutic and/or therapeutic conduct of the studied cases; critical review of the literature; critical review of the manuscript.

Sara Ibarbia-Oruezabal: Approval of the final version of the manuscript; design and planning of the study; drafting and editing of the manuscript; collection, analysis, and interpretation of data; effective participation in research orientation; intellectual participation in the propaedeutic and/or therapeutic conduct of the studied cases; critical review of the literature; critical review of the manuscript.

Nerea Segués-Merino: Approval of the final version of the manuscript; design and planning of the study; drafting and editing of the manuscript; collection, analysis, and interpretation of data; effective participation in research orientation; intellectual participation in the propaedeutic and/or therapeutic conduct of the studied cases; critical review of the literature; critical review of the manuscript.

## Conflicts of interest

None declared.
